# Clinical Significance of Candida in an Intraoperative Peritoneal Specimen with Perforation Peritonitis: An Institutional Perspective

**DOI:** 10.7759/cureus.2275

**Published:** 2018-03-05

**Authors:** Jagannath Pramod, Chellappa Vijayakumar, Krishnamachari Srinivasan, Nanda Kishore Maroju, Nagarajan Raj Kumar, Gopal Balasubramaniyan

**Affiliations:** 1 Surgery, Jawaharlal Institute of Postgraduate Medical Education and Research (JIPMER), Puducherry, India.

**Keywords:** fungal infection, morbidity, mortality, surgical site infections, quality of life, prognosis, peritonitis, peritoneal lavage, candida colonization, anti-fungal property

## Abstract

Introduction

Fungal infection of the peritoneum has become more common in recent years, the most common cause of which is Candida. Candida peritonitis is considered as a severe disease and is regarded as an independent risk factor for mortality in postoperative peritonitis. This study was planned to find out the clinical significance of Candida isolation on the outcome of the patients with peritonitis in terms of morbidity and mortality.

Methods

This prospective study included consecutive patients admitted and operated for secondary peritonitis over a two-year period in a tertiary care hospital in South India. The time delay was assessed from the onset of symptoms to surgery. The intraoperative peritoneal fluid aspirate was analyzed for culture sensitivity (fungal and bacterial). Patients were followed until their discharge from the hospital or death. This study analyzed the clinico-microbiological profile in patients with perforation peritonitis with special reference to Candida isolation. The analysis also looked the results of antifungal therapy (fluconazole) in patients positive for Candida isolation.

Results

The study included 407 consecutive patients with hollow viscus perforation diagnosed intraoperatively. Fungal organisms were identified in 153 patients (37.6%). Old age (> 50 years), high lag period (≥ 48 hours), peritoneal contamination, length of hospital stay, the presence of co-morbidities, shock at presentation, and postoperative complications were found to be significantly associated with fungal infection (p < 0.05). The study noted a significant decrease in the perioperative complications in patients who were started on antifungal treatment early (within 72 hours after surgery). There were significant reductions in the length of hospital stay, intensive care unit (ICU) stay, ventilator support, and inotropic support in the postoperative period. However, we did not find any difference in mortality due to early treatment with fluconazole.

Conclusion

Candida peritonitis was associated with an increase in the mortality and morbidity, especially when associated with diabetes mellitus and fungemia. Early antifungal therapy (within 72 hours after surgery) reduced the morbidity due to Candida peritonitis but did not affect the mortality.

## Introduction

Peritonitis is a common emergency encountered in surgical practice. Secondary peritonitis is the most common form of peritonitis [1]. It is most commonly due to perforation of a hollow viscus. Although the predominant pathogens involved in secondary peritonitis are gram-negative and anaerobic bacteria, the incidence of the fungal infection, especially in the critically ill, has increased in recent years [2]. Differences have been reported in the microbiological profile of the community-acquired and postoperative peritonitis. Enterococci are more common in postoperative peritonitis and yeasts are more commonly isolated in community-acquired cases [3].

It is recommended that the anti-microbial therapy should cover both anaerobes and gram-negative aerobes [4].The specific treatment aimed at Enterococci and Candida remains controversial [5]. An integral part of the surgical procedure involves the collection of peritoneal fluid for bacterial culture, based on which the antibiotics can be changed accordingly. In cases where host defenses are overwhelmed, diffuse peritonitis ensues [6].

Candida species are ubiquitous fungi that represent the most common fungal pathogens that affect humans. These are pervasive pathogens capable of causing both local and systemic infections in hospitalized patients [7]. Pathological fungal colonization is associated with multiple organ failure and high mortality [8]. Isolation of Candida from the peritoneal fluid is associated with high death rates [9]. Despite advances in medical technology and the development of new antifungal drugs, the crude and attributable mortality of candidemia has remained unchanged in the past 20 years [10]. This study was done to analyze the clinical outcome of patients started on antifungal therapy with a Candida infection of the peritoneal fluid.

## Materials and methods

This prospective clinical study was conducted for two years in a tertiary care hospital, in South India. All consecutive patients admitted and operated with a diagnosis of peritonitis secondary to hollow viscus perforation were included in this study. This study excluded patients with primary peritonitis, traumatic perforation, patients on antifungal treatment, and patients with perforation managed conservatively. The poor general condition of a few patients was the reason for a delay in surgery. These patients underwent bilateral preoperative flank drain placement (n = 16), and the fluid was sent for fungal culture and sensitivity. Such patients who succumbed to the disease (n = 16) were excluded from analysis. The time delay from the onset of symptoms to surgery was recorded. After the initial resuscitation, patients were scheduled for emergency exploratory laparotomy. The intraoperative findings, which were recorded, included the size of the perforation, the amount and the nature of the contamination, and the intraoperative hemodynamic stability.

During laparotomy, as soon as the peritoneum was opened, the peritoneal fluid specimen was taken in a sterile manner and was sent for gram stain, KOH (potassium hydroxide) mount, aerobic culture sensitivity, and fungal culture sensitivity. A sample of blood and urine were also collected for fungal culture and sensitivity. The Mannheim’s Peritonitis Index (MPI) was calculated for each patient to assess the risk stratification. The sensitivity of the fungal isolates, especially Candida, was determined for fluconazole and amphotericin B.

All patients received the same antibiotics which were started at admission and continued into the postoperative period. The initial choices of the antibiotics were according to the institute protocol and relevant changes of antibiotics were carried out according to the culture and sensitivity report. In the postoperative period, the need for mechanical ventilation, inotropic support, and stay in the Intensive Care Unit (ICU) were recorded. The postoperative complications recorded in the study were surgical site infection (SSI), wound dehiscence/burst abdomen, intra-abdominal collection/abscess, enteric leak, respiratory complications, and septicemia. In cases where patients had an SSI, the wound swab was sent for culture and sensitivity and the microbiological profile was recorded. The patients who had an intra-abdominal abscess/collection underwent ultrasonography-guided aspiration and the aspirate was sent for culture and sensitivity.

**Figure 1:**
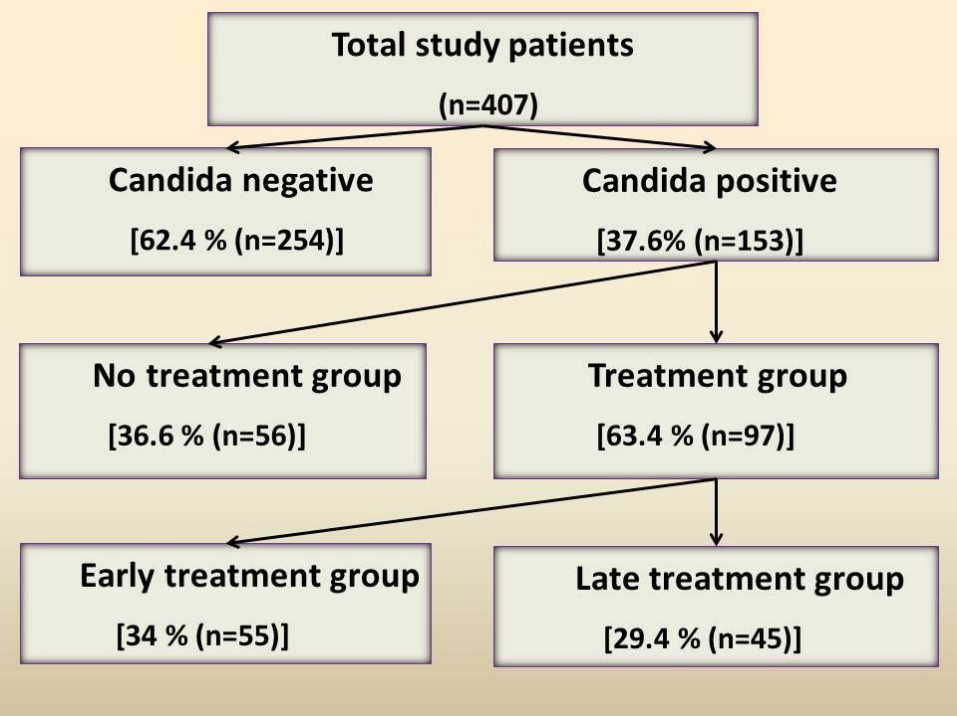
Study Flowchart

According to the study protocol, the patients who were found to have Candida detected in their peritoneal fluid or blood or urine by any mode were to be started on antifungal therapy with fluconazole. The dose of the drug (fluconazole) used was 200 mg administered twice daily intravenously until the resolution of the postoperative ileus and then changed over to oral or enteral fluconazole tablets, 150 mg twice a day. The total duration of treatment was 14 days. Based on the timing of the start of fluconazole therapy, these patients (Candida-positives) were divided into two groups - the early treatment group (within 72 hours of surgery) and the late treatment group (beyond 72 hours of surgery). In addition, about one-third of the patients who had Candida detected could not be started on antifungal therapy with fluconazole as the patient had expired or had been discharged from the hospital before the availability of the culture reports. This group of Candida-positive patients who could not be started on therapy was called the “No treatment group” (Figure 1).

The perioperative parameters, including mortality, were compared between the Candida-positive/negative groups to assess the effect of Candida on the outcome of the patient. Again, these parameters were compared four weeks postoperatively between the early treatment, late treatment, and the no treatment groups in order to assess the efficacy of fluconazole in Candida-positive patients.

The statistical analysis was performed using the Statistical Package for Social Sciences (SPSS) 16.0 software (IBM SPSS Statistics, Armonk, NY). The Chi-square test or the Fisher’s exact test was performed to determine the significance of association in non-parametric data between the groups. For comparing the means of the parametric variables, the Student’s t- test was performed. A multivariate analysis using stepwise logistic regression was performed to determine the significance of Candida in the outcome of the patient. A probability of < 0.05 was considered significant.

## Results

Perioperative parameters

This study included 339 males (83.3%) and 68 females (16.7%). The mean difference in age between the Candida-positive and negative groups was statistically significant (46.5 vs. 42.8; p = 0.01). Among the 407 patients, the mean duration of the symptoms between the groups was (37.0 vs. 44.3; p = < 0.0001), which was found to be statistically significant. The mean time lapse from admission to laparotomy between the two groups was statistically significant (45.3 vs. 53.1; p = 0.002). The number of patients with one or more comorbid illnesses between the groups was significant (46 vs. 65; p = 0.0001). The incidence of Candida-positive peritonitis was found to be more in diabetics (40 vs. 18; p = 0.001). There was no statistically significant association between any of the other comorbidities and Candida infection. Ninety-two patients (22.6%) presented to the casualty in shock. The study also found a significant association of Candida with a higher amount of peritoneal contamination (1,609 vs. 1,865; p = 0.0001) and larger size of the perforation (5.08 vs. 5.83; p = 0.03), probably as a consequence of the delayed presentation in this group of patients. A higher proportion of patients in the Candida-positive group had perioperative hemodynamic-related problems and, as a result, required mechanical ventilation and inotropic support more often and for a longer duration compared to the Candida-negative group (Table 1).

**Table 1:** Comparison of Perioperative Parameters Between Candida-positive/negative Groups N: number; SSI: surgical site Iinfection; DM: diabetes mellitus; ECF:  enterocutaneous fistula; RS: respiratory system

Perioperative parameters	Candida negative (n = 254)	Candida positive (n = 153)	p - value
Preoperative			
Mean age (years)	42.8	46.5	0.01
Mean delay (hrs)	45.3	53.1	0.0002
Comorbidity (N (%))	46 (18.1%)	65 (42.5%)	0.0001
DM (N (%))	18 (7.1%)	40 (26.1%)	0.003
Shock (N (%))	39 (15.4%)	53 (34.6%)	0.00
Intraoperative			
Mean perforation (mm)	5.08	5.83	0.03
Mean contamination (ml)	1,609	1,865	0.0001
Postoperative			
Mean hospital stay (days)	13.2	18.1	0.001
SSI (N (%))	102 (66.7%)	126 (49.6%)	0.001
Dehiscence (N (%))	44 (28.8%)	32 (12.6%)	0.001
Abscess (N (%))	36 (23.5%)	37 (14.6%)	0.022
ECF (N (%))	28 (18.3%)	14 (5.5%)	0.001
RS complications (N (%))	56 (36.6%)	50 (19.7%)	0.001

Fungal culture of the peritoneal fluid

The fungal culture of the intraoperative peritoneal fluid turned out to be positive in 153 patients (37.5%). In the Candida-positive group, C. albicans was the most frequently detected species (44 cases, 35.5%). In the Candida-positive group, there were 90 patients (58.8%) who were positive for a fungal culture of the peritoneal fluid. However, in these patients, Candida was detected by at least one of the other methods of identification. The basis for starting these patients on antifungal treatment was either microscopy or aerobic culture reports (Table 2).

**Table 2:** Basis of Starting Antifungal Treatment in Candida-positive Patients N: number; KOH: potassium hydroxide

Basis for starting fluconazole (n = 153)	N (%)
Gram stain of peritoneal fluid	3 (2%)
KOH mount of peritoneal fluid	32 (20.9%)
Aerobic culture of peritoneal fluid	17 (11.1%)
Fungal culture of peritoneal fluid	90 (58.8%)
Fungal culture of blood	7 (4.6%)
Fungal culture of urine	4 (2.6%)

Antifungal treatment

Of the 97 patients (63.4%) who were started on antifungal therapy, fluconazole was started within 72 hours after surgery (early treatment group) in 52 patients (34%) and the rest of them (45 cases, 29.4%) received therapy started after 72 hours following surgery (late treatment group) (Table 3).

**Table 3:** Treatment-based Categorization of Candida-positive Patients N: number; KOH: potassium hydroxide; Tx: treatment

Category	Basis (n = 153)	N (%)
No Tx group	Patient’s expiry or discharge before reporting	56 (36.6%)
Tx group	Early + late Tx	97 (63.4%)
Early Tx group (< 72 hrs. after surgery)	Gram stain/KOH mount/aerobic culture report	52 (34%)
Late Tx group (> 72 hrs. after surgery)	Fungal culture of peritoneal fluid/blood/urine report	45 (29.4%)

MPI score and its association with mortality

On comparing the mean MPI score between the Candida-positive/negative groups, it was found that the mean (22.87 vs. 25.20; p = 0.004) difference was statistically significant. There was no statistically significant difference between the means of the MPI score in the antifungal untreated group (24.8 vs. 25.4; p = 0.53) and the treated group. There was a high mortality (54.7%) in patients with MPI scores more than 29 (Table 4).

**Table 4:** Mannheim’s Peritonitis Index (MPI) Score and Its Association with Mortality in Candida-positive/negative Groups N: number

	Candida-negative		Candida-positive		Total
MPI score	N	Mortality (N (%))	N	Mortality (N (%))	(N (%))
< 21 (n = 193)	140	5 (21.7%)	53	2 (6.7%)	7 (13.2%)
22 – 29 (n = 125)	64	4 (17.4%)	61	13 (43.3%)	17 (32.1%)
> 29 (n = 89)	50	14 (60.9%)	39	15 (50%)	29 (54.7%)
Total (n = 407)	254	23 (9%)	153	30 (19%)	53 (13%)

Postoperative complications between the early/late treatment groups

The complications that occurred in the early postoperative period (less than one month) were significantly higher in the Candida-positive group than in the Candida-negative group. Among the Candida-positives, the rate of complications seemed to be more in the antifungal treated category than in the antifungal untreated category. It was also noted that all of the late complications were significantly lesser in the early treatment group when compared with the late treatment group. Thus, the overall mortality rate in the study was 13% (53 cases). Among them, 30 patients (56.6%) were positive for Candida and 23 patients (23 cases, 43.39%) were negative for Candida. There were nine mortalities (16.1%) in the antifungal untreated category and 21 (19.6%) in the antifungal treated category. In the early antifungal treatment group, 12 patients (23.1%) succumbed to death, while nine patients (20%) did so in the late treatment group (p = 0.71) (Table 5).

**Table 5:** Comparison of Postoperative Complications Between the Early/Late Treatment Groups N: number; SSI: surgical site infection; ICU: Intensive Care Unit; ECF: enterocutaneous fistula; RS: respiratory system; Tx: treatment

Clinical Parameters	Early Tx group (n = 52)	Late Tx group (n = 45)	p - value
Mean ventilator support (Days)	1.2	3.3	0.025
Mean inotropic support (Days)	0.4	2.2	0.005
Mean ICU stay (Days)	3.9	9.2	0.001
Mean hospital Stay (Days)	13.9	31.6	0.00
SSI (N (%))	36 (69.2%)	41 (91.1%)	0.008
Dehiscence (N (%))​​​​​​​	12 (23.1%)	25 (55.6%)	0.001
Abscess (N (%))​​​​​​​	11 (21.2%)	15 (33.3%)	0.17
ECF (N (%))​​​​​​​	6 (11.5%)	17 (37.8%)	0.002
RS complications (N (%))​​​​​​​	18 (34.6%)	29 (64.4%)	0.003
Septicemia (N (%))​​​​​​​	14 (26.9%)	22 (48.9%)	0.026
Mortality (N (%))​​​​​​​	12 (23.1%)	9 (20%)	0.71

Perioperative complications between treated and untreated groups

The mean age of the patients in the untreated (without antifungal) group was not significant when compared with treated (with antifungal) group (46.29 vs. 46.09; p = 0.6). The mean duration of symptoms in the early and late treatment groups were comparable (49.5 vs. 50.5; p = 0.38). Comparison between the early and late treatment categories with regard to the complications at one month after surgery showed a statistically significant decrease in the occurrence of SSI (p = 0.003), wound dehiscence (p = 0.002), septicemia (p = 0.005), respiratory complications (p = 0.001), and enterocutaneous fistula (p = 0.001) in the early antifungal treatment group. Mortality was high (9 vs. 21; p = 0.40) in no treatment group but the difference was not significant (Table 6).

**Table 6:** Comparison of Perioperative Parameters Between Untreated and Treated Groups N: number; Tx: treatment; SSI: surgical site infection; DM: diabetes mellitus; ECF:  enterocutaneous fistula; ICU: Intensive Care Unit; S: Sterile; -: negative; +: positive; RS: respiratory system

Perioperative parameters	No Tx group (n = 56)	Tx group (n = 97)	p - value
Preoperative			
Mean age (years)	46.3	46.09	0.60
Mean duration of symptoms (hours)	40.3	46.6	0.04
DM (N (%))	14 (25%)	26 (26.8%)	0.81
Shock (N (%))	10 (17.9%)	43 (44.3%)	0.001
Intraoperative			
Mean contamination (ml)	1,675	1,974	0.003
Hemodynamic instability (N (%))	9 (16.1%)	26 (26.8%)	0.13
Postoperative			
Mean ICU stay (days)	3.2	6.4	0.006
Mean hospital stay (days)	11.0	22.1	0.00
Fluid culture^s^ (N (%))	19 (33.9%)	23 (23.7%)	0.24
Fluid culture^-^ (N (%))	2 (3.6%)	27 (28.9%)	0.00
Blood culture^+^ (N (%))	13 (23.25%)	39 (40.2%)	0.03
Urine culture^+^ (N (%))	18 (32.1%)	48 (49.5%)	0.03
SSI (N (%))	25 (44.6%)	77 (79.4%)	0.003
Dehiscence (N (%))	7(12.5%)	37 (38.1%)	0.002
Abscess (N (%))	10 (17.9%)	26 (26.8%)	0.209
ECF (N (%))	5 (8.9%)	23 (23.7%)	0.02
RS complications (N (%))	9 (16.1%)	47 (48.5%)	0.001
Septicemia (N (%))	11 (19.6%)	36 (37.1%)	0.005
Mortality (N (%))	9 (16.1%)	21 (21.6%)	0.40

Impact of fungemia after a one-month postoperative period

More patients with hemodynamic compromise qualified for antifungal therapy. This could have been a contributory factor for higher mortality and morbidity in the category of patients who received antifungal therapy. Length of ICU stay, shock at admission, and Candida-positive blood/urine culture were significant risk factors for both respiratory complications and septicemia. The significant factors influencing mortality were the duration of symptoms at presentation, lapse period, shock at admission, MPI score, septicemia, and enteric leak. Overall complications were high in the no-treatment and late treatment groups (Table 7).

**Table 7:** Impact of Antifungal Treatment on Complications After One Month of Postoperative Period in Candida-positive/negative Groups SSI: surgical site infection; N: number; Tx: treatment; RS: respiratory system; ECF: enterocutaneous fistula

Complications	Candida negative (N (%))	Candida positive (N (%))			
		No Tx	Early Tx	Late Tx	Total
SSI	66 (36.5%)	9 (23.1%)	14 (43.8%)	34 (85%)	57 (51.4%)
Dehiscence	23 (12.7%)	2 (5.1%)	6 (18.8%)	23 (57.5%)	31 (27.9%)
Septicemia	4 (2.2%)	-	1 (3.1%)	13 (32.5%)	14 (12.6%)
RS complications	3 (1.7%)	-	-	13 (32.5%)	13 (11.7%)
ECF	3 (1.7%)	-	1 (3.1%)	10 (25%)	11 (9.9%)
Overall complications	115 (63.5%)	30 (76.9%)	18 (56.2%)	34 (85%)	54 (48.7%)

## Discussion

In our study, the incidence of intraperitoneal fungal infection was 37% and that of Candida was 30%. The reported incidence of Candida peritonitis was around 3 - 12% [8]. The incidence of intraperitoneal fungal infection was 43.4% in the review of patients with perforated peptic ulcer done by Shan et al. [9]. In accordance with the recent recommendations by the Infectious Disease Society of America, fluconazole therapy was started as soon as Candida was identified in the peritoneal fluid microscopy or culture [10]. We also assessed the impact of treatment on the outcome of the patients, age, and gender distribution. Our study included a total of 407 patients with a mean age of 44.2 was comparable with the previous studies done in perforation peritonitis by Arveen et al. (43.4 years) [11]. Shan et al. reported that fungal infection was more common in males (48%). However, in this study, Candida infection was detected more among females (47%) than males (35%) [9].

Preoperative factors

This study also noted a longer duration of symptoms and a delay in presentation with shock in the fluconazole-treated group of patients among the Candida-positives. This may, in part, explain the fallacious increase in the complications in this group. A longer time lapse from the onset of symptoms to the laparotomy was associated with a higher chance of Candida detection, which might be due to the time required for the Candida to multiply in number and become detectable more often. The late presentation to the hospital has been reported in the previous studies to be associated with an increase in the mortality rate [11]. This association was noted in our study as well. Our study noted a statistically significant increase in the incidence of Candida peritonitis in diabetics. This was in contrast to the study results of the National Epidemiology of Mycosis Survey (NEMIS) trial where they found renal failure to be a significant risk factor for Candida infection [12]. In this study, the incidence of septic shock was noticed more often in patients with delayed presentation (> 48 hours) (75% vs. 15%). This was in sharp contrast to a prior study by Arveen et al. [11]. Shock explains the higher mortality rate noted in this subset of patients as compared with the untreated group.

Intraoperative factors

As documented in many of the other studies, the incidence of duodenal perforation was high in this study as well [13]. We did not find any significant relationship between the type of soiling and the Candida infection. However, the Candida-positive group had an increased amount of contamination, which can be explained in terms of the delay in the presentation. In this study, they found a significant association with the size of the perforation and Candida positivity. This may be due to the delay in the presentation of these patients, providing time for the growth and multiplication of Candida intra-abdominally. It was this set of patients who presented with shock more than the others and needed preoperative inotropic and mechanical ventilator support. They also noted the higher proportion of hemodynamic instability in the Candida-positive group. It may be again explained by the late presentation of these patients.

Fungal culture of the peritoneal fluid

The positivity of fungal cultures was 43.4% in a study of 144 peptic ulcer patients done by Shan et al. [9]. This could probably be due to the fact that Candida is more common in the upper gastrointestinal pathology and this study included both upper and lower GI perforations. Candida infection was never found in isolation.

MPI scoring system

There was a significant increase in mortality with an increase in the MPI score. The corresponding mortality in a similar study done by Shan et al. was zero, 7%, and 80% [9]. This study substantiates the fact that a higher MPI score was associated with a poor prognosis. The presence of a high MPI score was one of the risk factors for fungal culture positivity identified by multivariate analysis in the study done by Shan et al. [9].

Postoperative complications

This study analysed postoperative complications that were significantly increased in the Candida-positive group when compared with the Candida-negative group. In most of the complications, Candida positivity on blood culture was found to be a significant factor influencing the morbidities. Moreover, none of the complications seemed to be influenced by the detection of Candida in the peritoneal fluid. The detection of Candida in the urine sample was a significant determinant of septicemic complications in the postoperative period. In his study, Shan et al. noted a higher incidence of SSI in patients positive for fungus in the peritoneal fluid, which correlate with the results of this study [9]. In the study by Lee et al., positive fungal culture for peritonitis was associated with a higher mortality and Candida co-infection was proposed to be a bad prognostic factor [14]. The results of this study also indicated a similarly poor prognosis associated with Candida peritonitis.

Mortality

The mortality rate was close to 50% in patients who underwent surgery after 72 hours of symptoms onset, whereas none of the 38 patients who underwent surgery within 24 hours expired. This study found a significant increase in the deaths in the Candida-positive group compared with the Candida-negative group. A similar finding was noted by Shan et al. and Lee et al. [9, 14]. Peritoneal fluid positivity for Candida was not a significant risk factor for mortality. However, the presence of fungemia turned out to be a significant risk for mortality. The other significant factors were the delay in the presentation, delay in performing a laparotomy, the presence of shock at admission, high MPI score, and enteric leak complication in the postoperative period.

Antifungal treatment

Current recommendations and guidelines emphasize the fact that antifungal therapy is not advocated as an empirical treatment option. These studies have demonstrated a decrease in the hospital mortality among critically ill patients at high risk of fungal sepsis [15]. On comparing the untreated cases with the treated cases, they noticed a fallacious statistically significant increase in the number of complications in the treated group than in the untreated group. Also, the mortality rate was noted to be higher, although insignificant, in the treated patients. This could possibly be due to the significant delay in presentation, a higher proportion of patients in shock at presentation, and/or a higher proportion of patients with diabetes in the treated group. Furthermore, the patients in the treatment group had more patients positive for Candida in the blood and urine.

The treatment of patients with antifungals was further justified by the fact that the early treatment group had significantly lower rates of complications (all except intra-abdominal abscess) compared with the late treatment group. There was also a significantly higher number of patients in the early treatment group with no complications in the follow-up period. However, even with all the benefits of reducing the morbidity, early treatment did not seem to influence the mortality compared with the late treatment group.

Follow-up

During one month follow-up, this study did not find any serious drug-induced complications and/or antifungal resistance in patients. Only a few patients had asymptomatic mild elevation serum transaminases, which did not require any active intervention. Thus, with the significant benefits and lack of serious adverse drug reactions, fluconazole was used as the first-line therapy for Candida infections. However, the Candida species detected may be invasive in some high-risk patients who are debilitated or immunocompromised. This group of patients may benefit from therapy, especially started early in the postoperative period.

Limitations

Postoperative complications were analysed among the patients only who were available for follow-up. Another limitation of this study was either late inclusion or non-inclusion of patients into the treatment category due to delay in the availability of fungal culture reports. This was due to the subtyping of Candida species, which required two to three weeks. However, in the latter part of the study, they could overcome this limitation by receiving reports even before subtyping. The patients were started on treatment based on the detection of Candida alone without waiting for speciation of the Candida.

## Conclusions

Candida could be identified in the majority of the patients with perforation peritonitis, of which C. albicans was the most common subtype. Candida peritonitis was associated with an increase in the mortality and morbidity, especially when associated with diabetes mellitus and fungemia. Early antifungal therapy (within 72 hours after surgery) reduced the morbidity due to Candida peritonitis but did not affect the mortality. With increasing incidence of fungal infections of the abdomen, there is a need to track the trends in order to establish guidelines for early diagnosis and management of these infections.
